# Supplementary figures and images for: Wattpad as a resource for literary studies. Quantitative and qualitative examples of the importance of digital social reading and readers’ comments in the margins
Source: PLoS One. 2020 Jan 15;15(1):e0226708. doi: 10.1371/journal.pone.0226708 (PMC6961871; doi:10.1371/journal.pone.0226708)

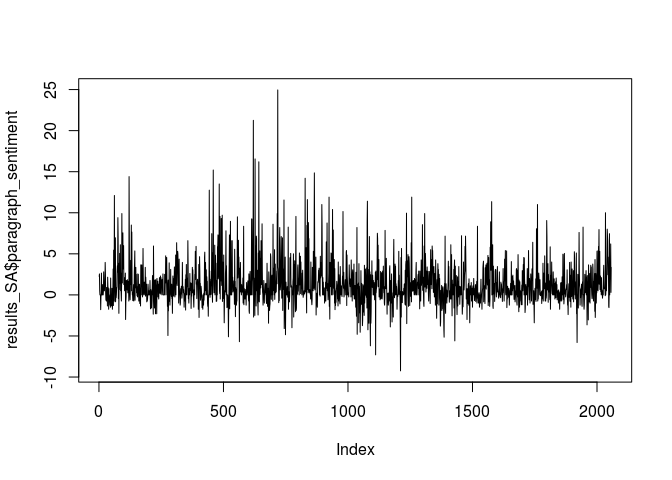

Supplement: S1 File — (ZIP) [file pone.0226708.s001.zip › Wattpad_analysis-1.0.0/Wattpad_sentiment_analysis_files/figure-markdown_github/unnamed-chunk-7-1.png]

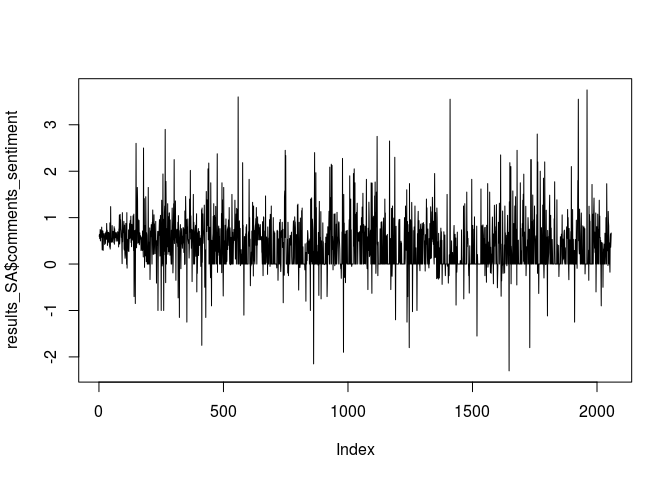

Supplement: S1 File — (ZIP) [file pone.0226708.s001.zip › Wattpad_analysis-1.0.0/Wattpad_sentiment_analysis_files/figure-markdown_github/unnamed-chunk-7-2.png]

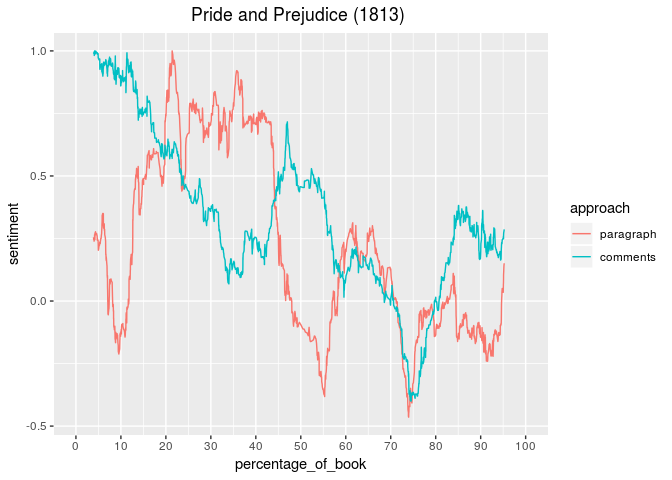

Supplement: S1 File — (ZIP) [file pone.0226708.s001.zip › Wattpad_analysis-1.0.0/Wattpad_sentiment_analysis_files/figure-markdown_github/unnamed-chunk-9-1.png]

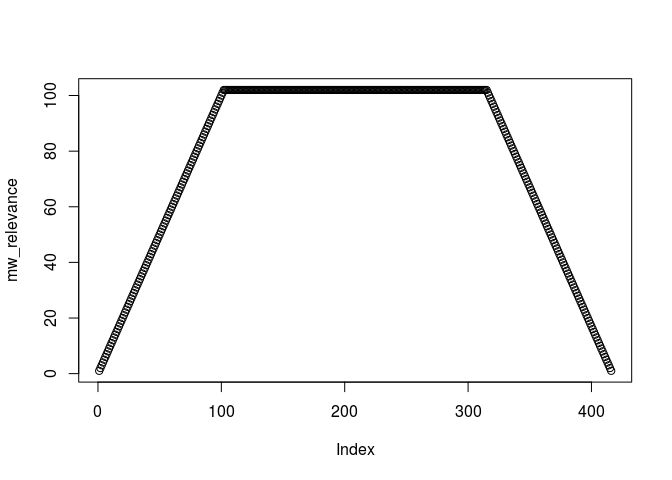

Supplement: S1 File — (ZIP) [file pone.0226708.s001.zip › Wattpad_analysis-1.0.0/reverse_engineering_syuzhet_files/figure-markdown_github/unnamed-chunk-5-1.png]

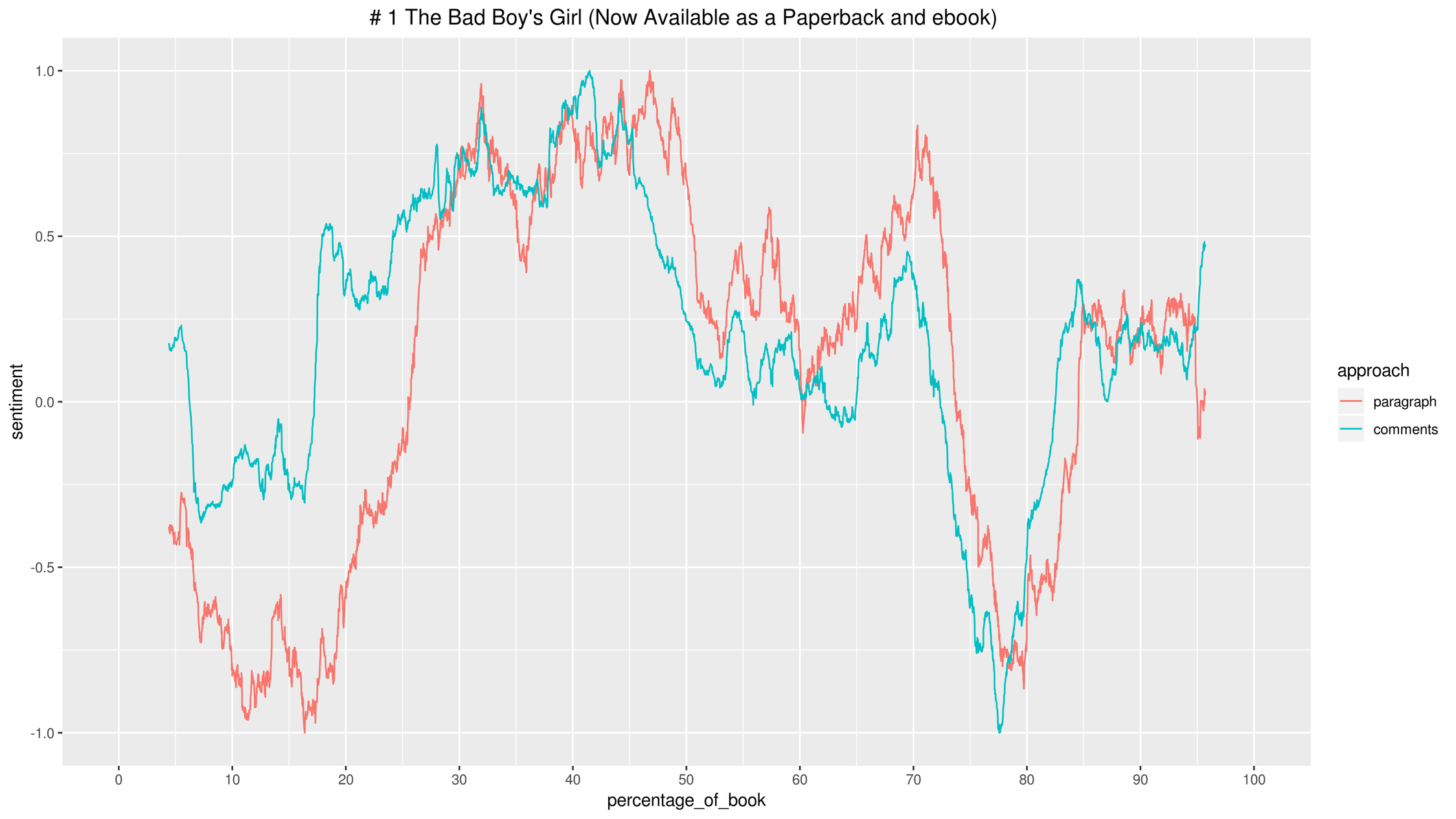

Supplement: S8 Fig — (TIF) [file pone.0226708.s013.tif]

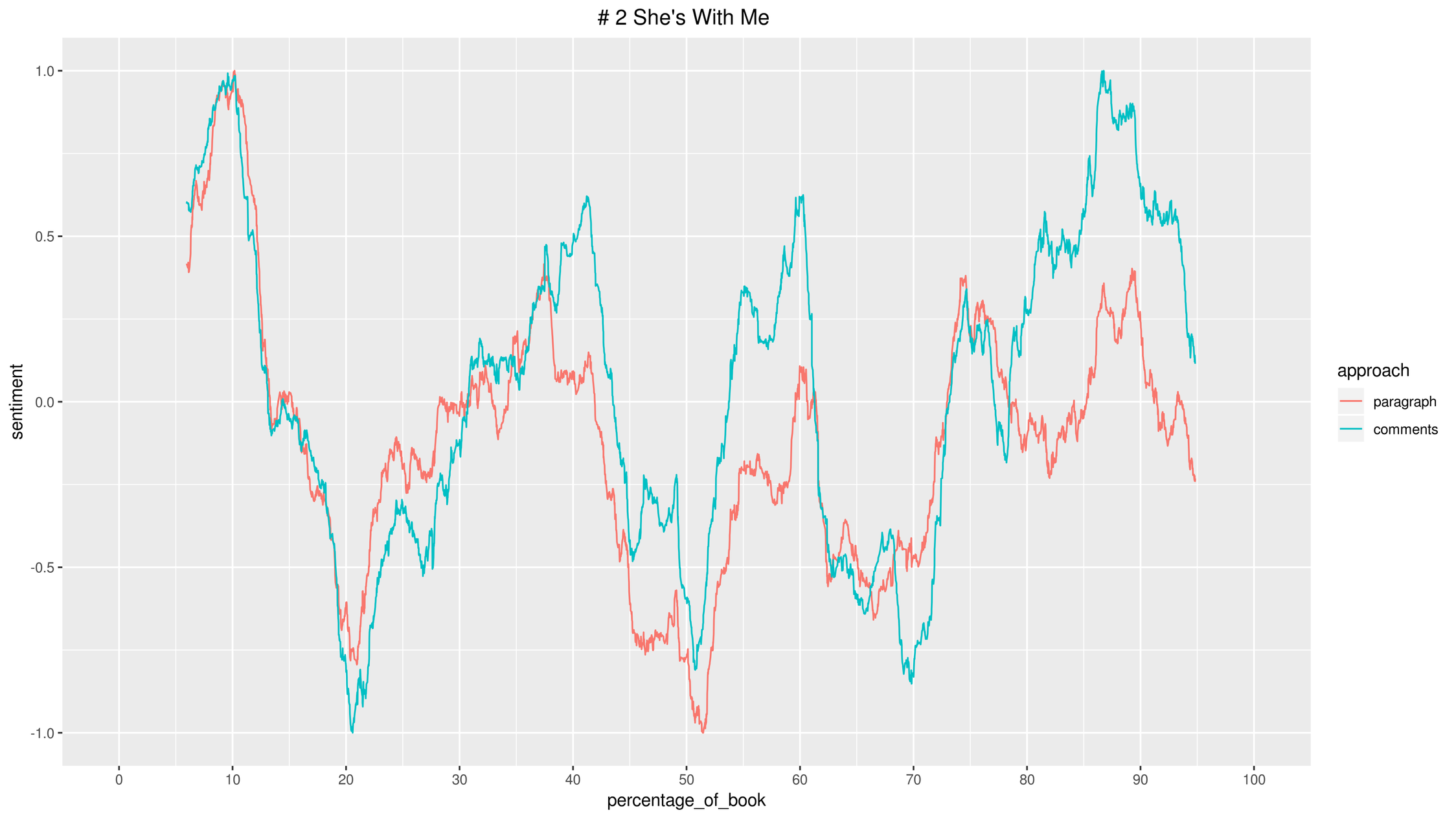

Supplement: S9 Fig — (TIF) [file pone.0226708.s014.tif]

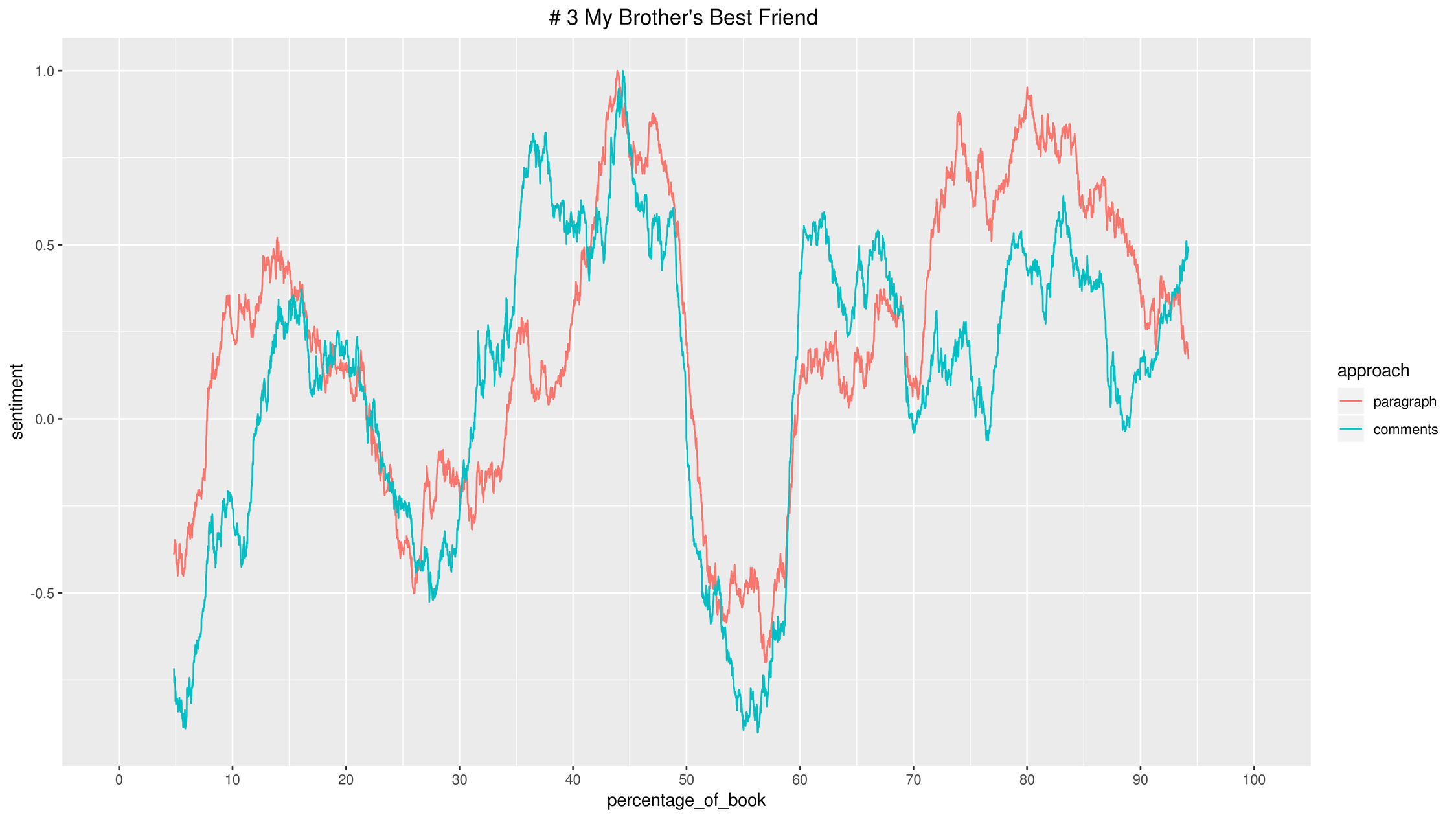

Supplement: S10 Fig — (TIF) [file pone.0226708.s015.tif]

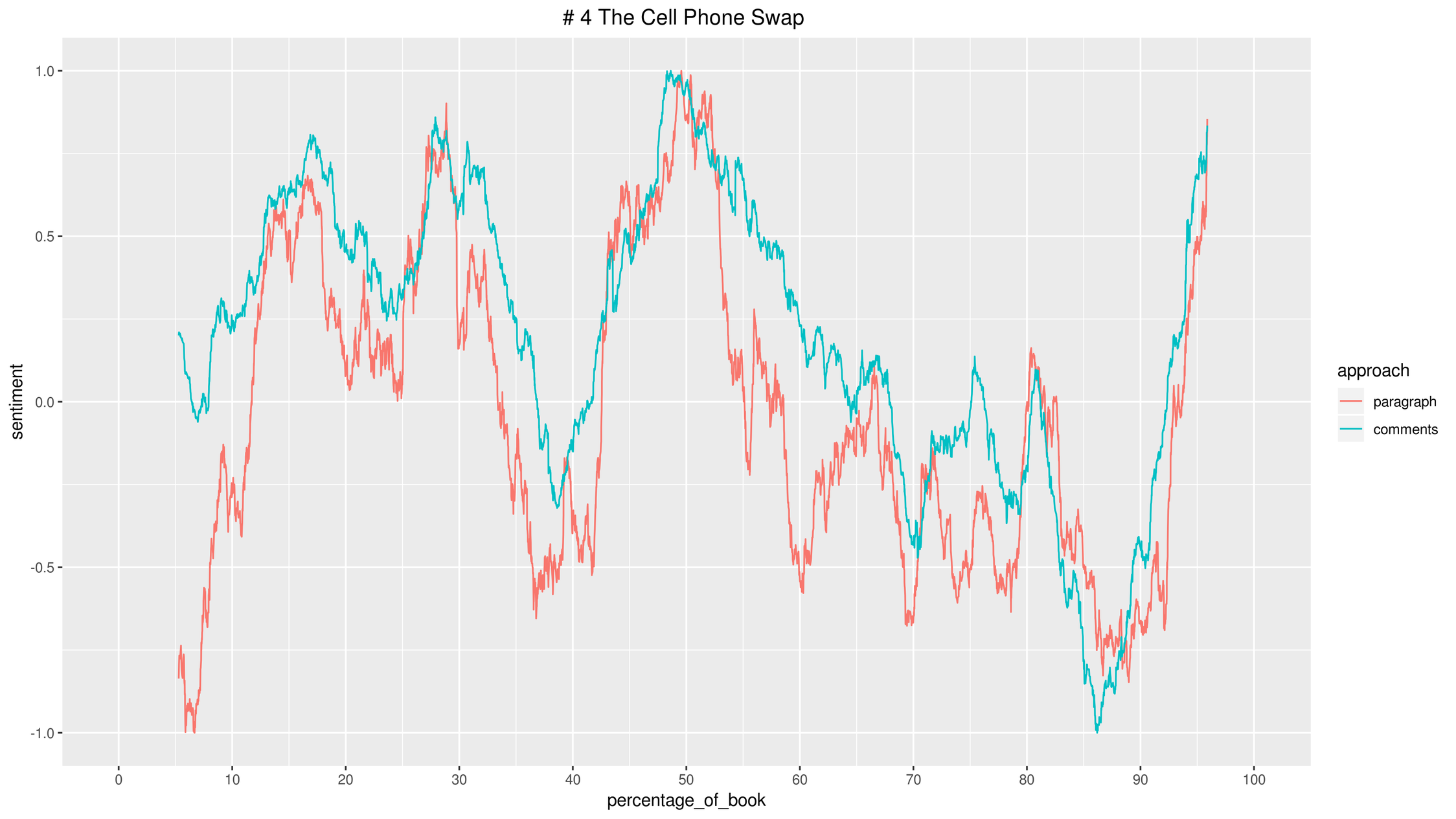

Supplement: S11 Fig — (TIF) [file pone.0226708.s016.tif]

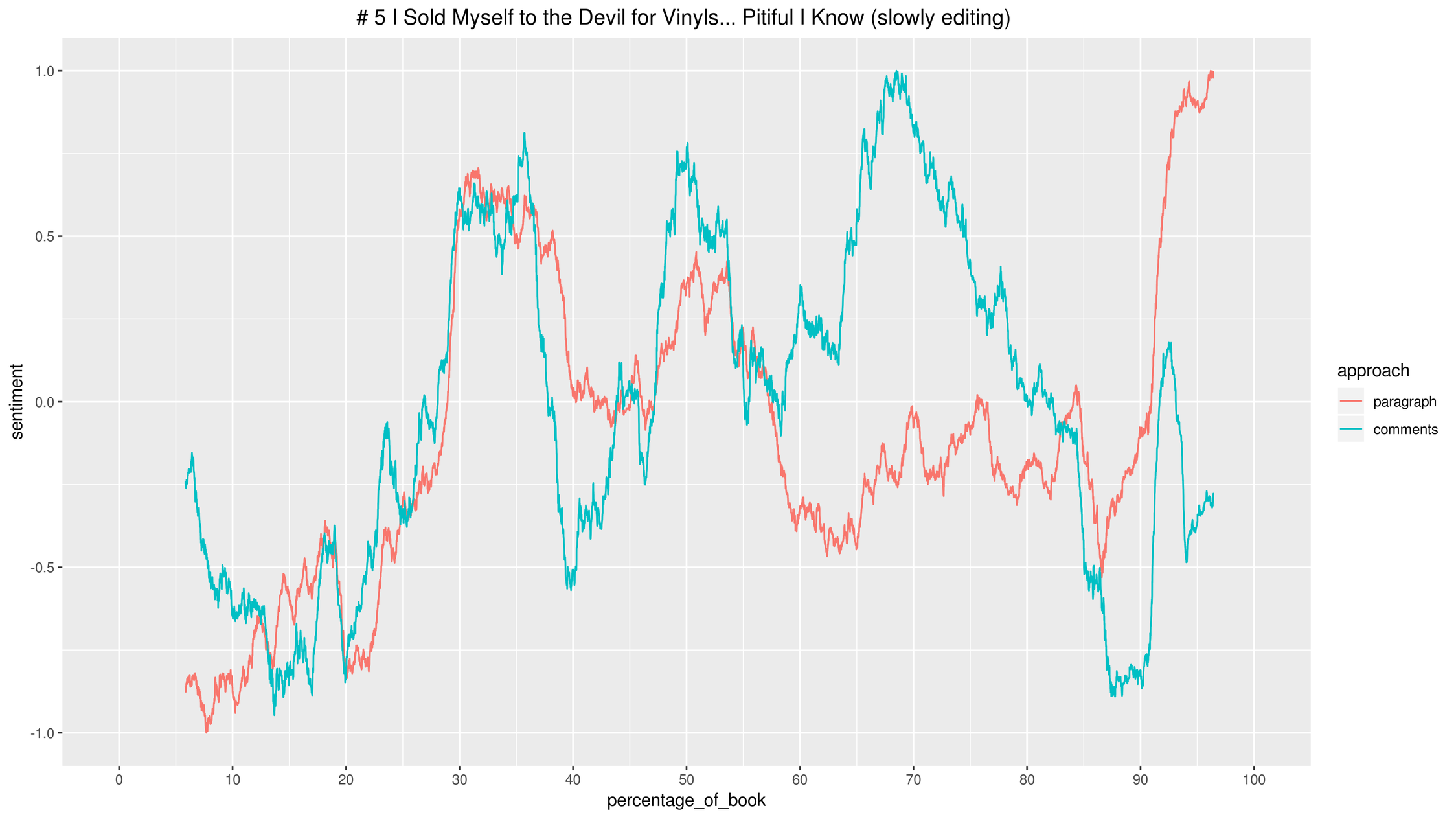

Supplement: S12 Fig — (TIF) [file pone.0226708.s017.tif]

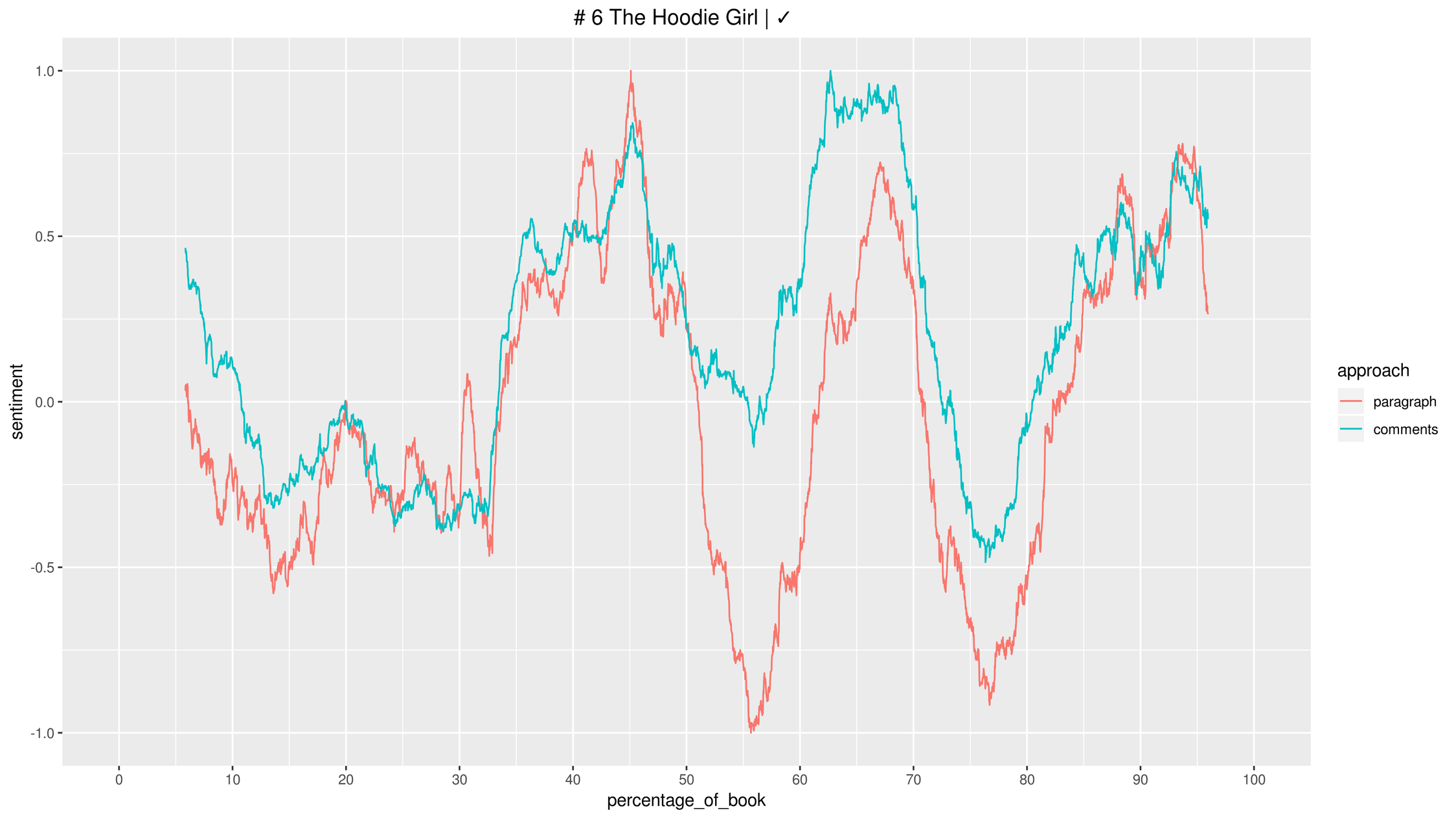

Supplement: S13 Fig — (TIF) [file pone.0226708.s018.tif]

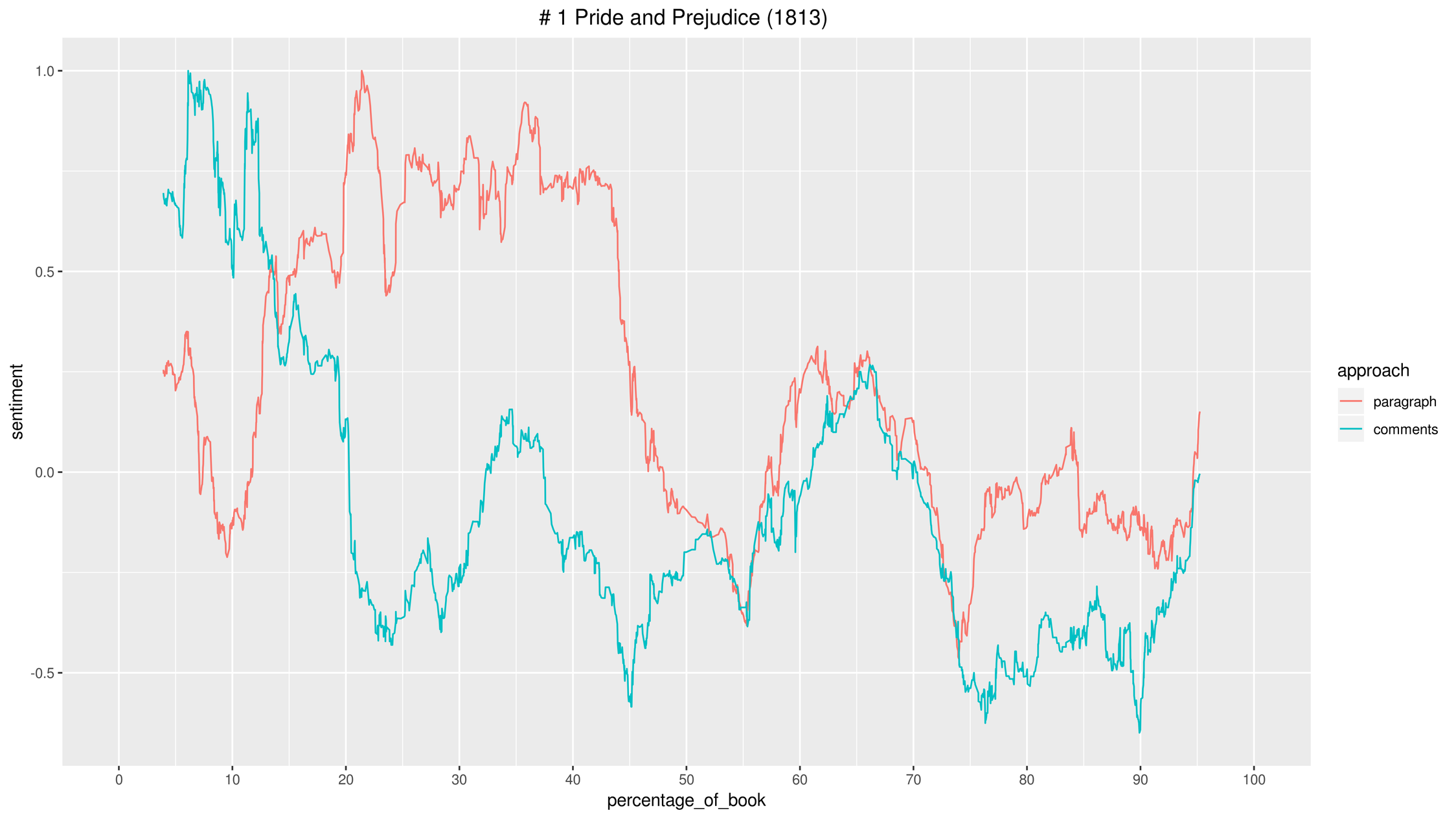

Supplement: S14 Fig — (TIF) [file pone.0226708.s019.tif]

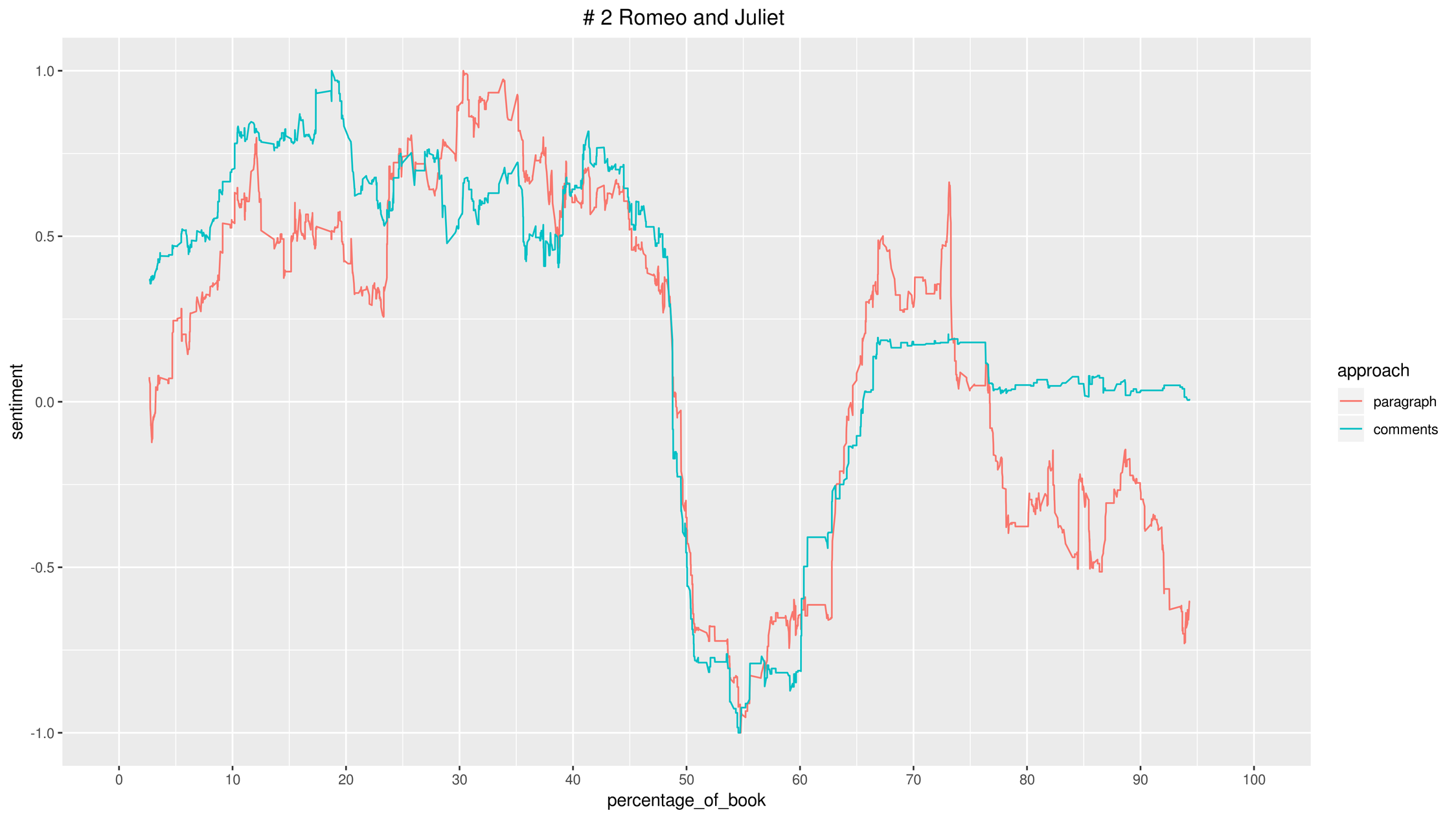

Supplement: S15 Fig — (TIF) [file pone.0226708.s020.tif]

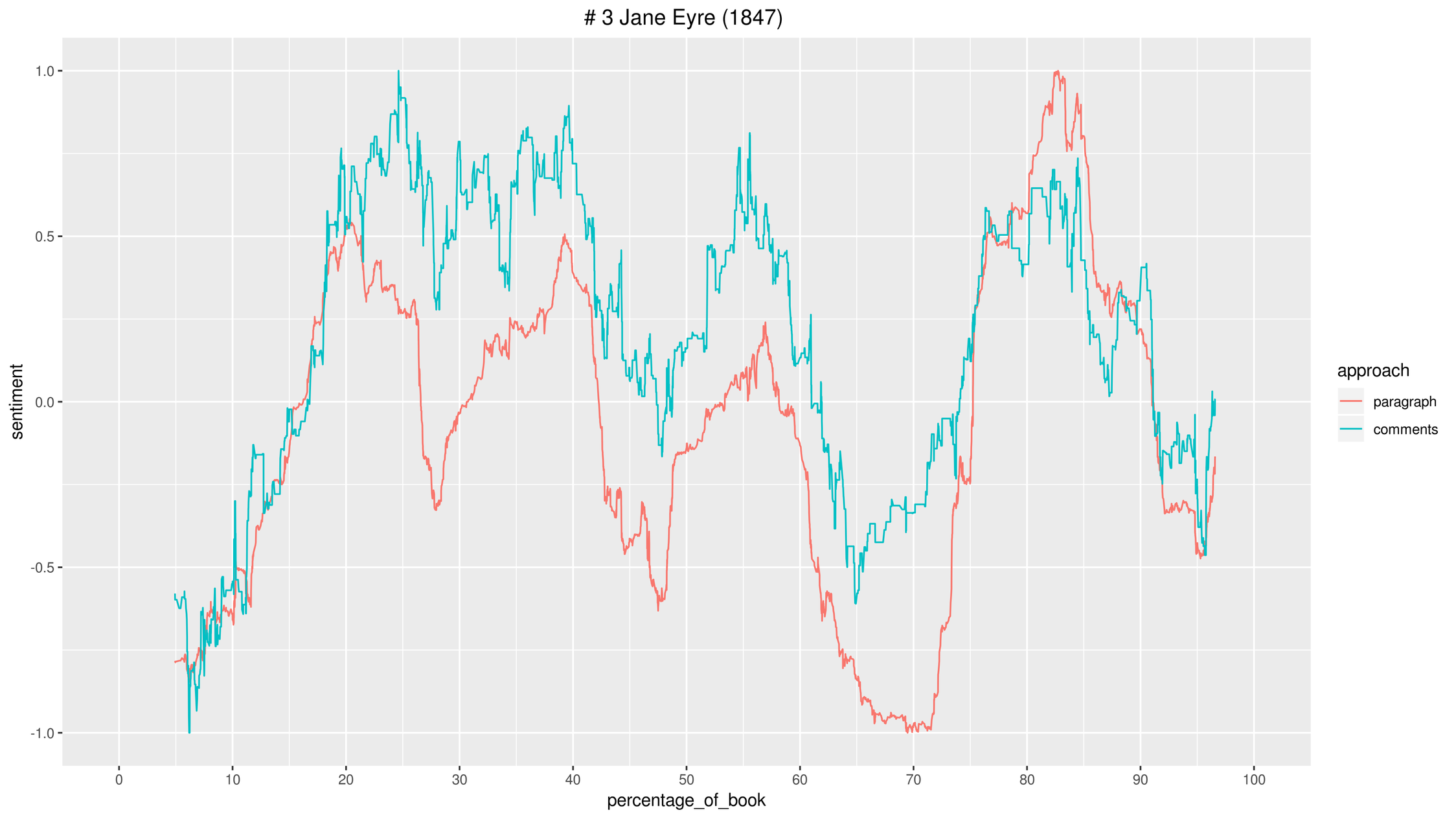

Supplement: S16 Fig — (TIF) [file pone.0226708.s021.tif]

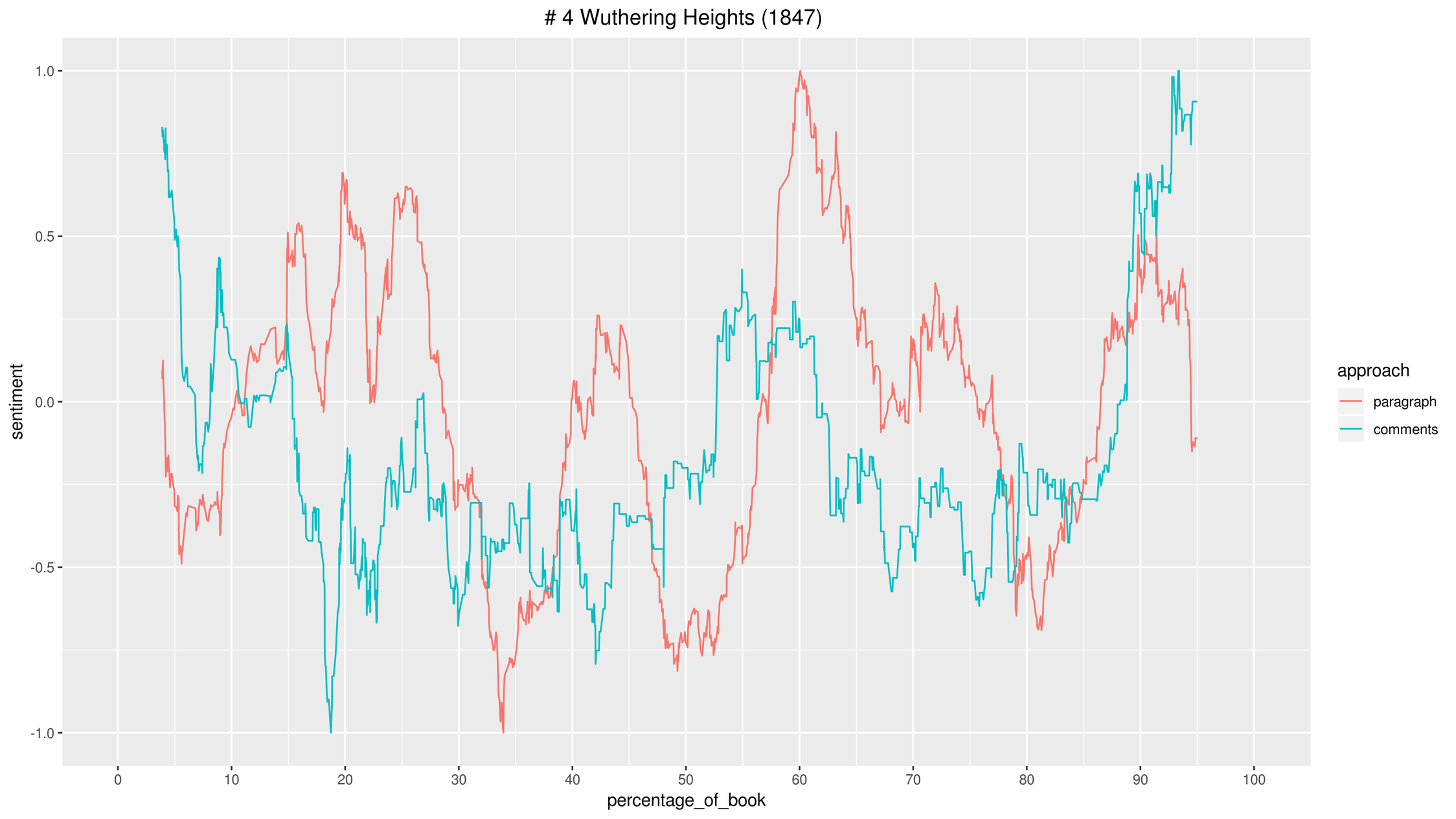

Supplement: S17 Fig — (TIF) [file pone.0226708.s022.tif]

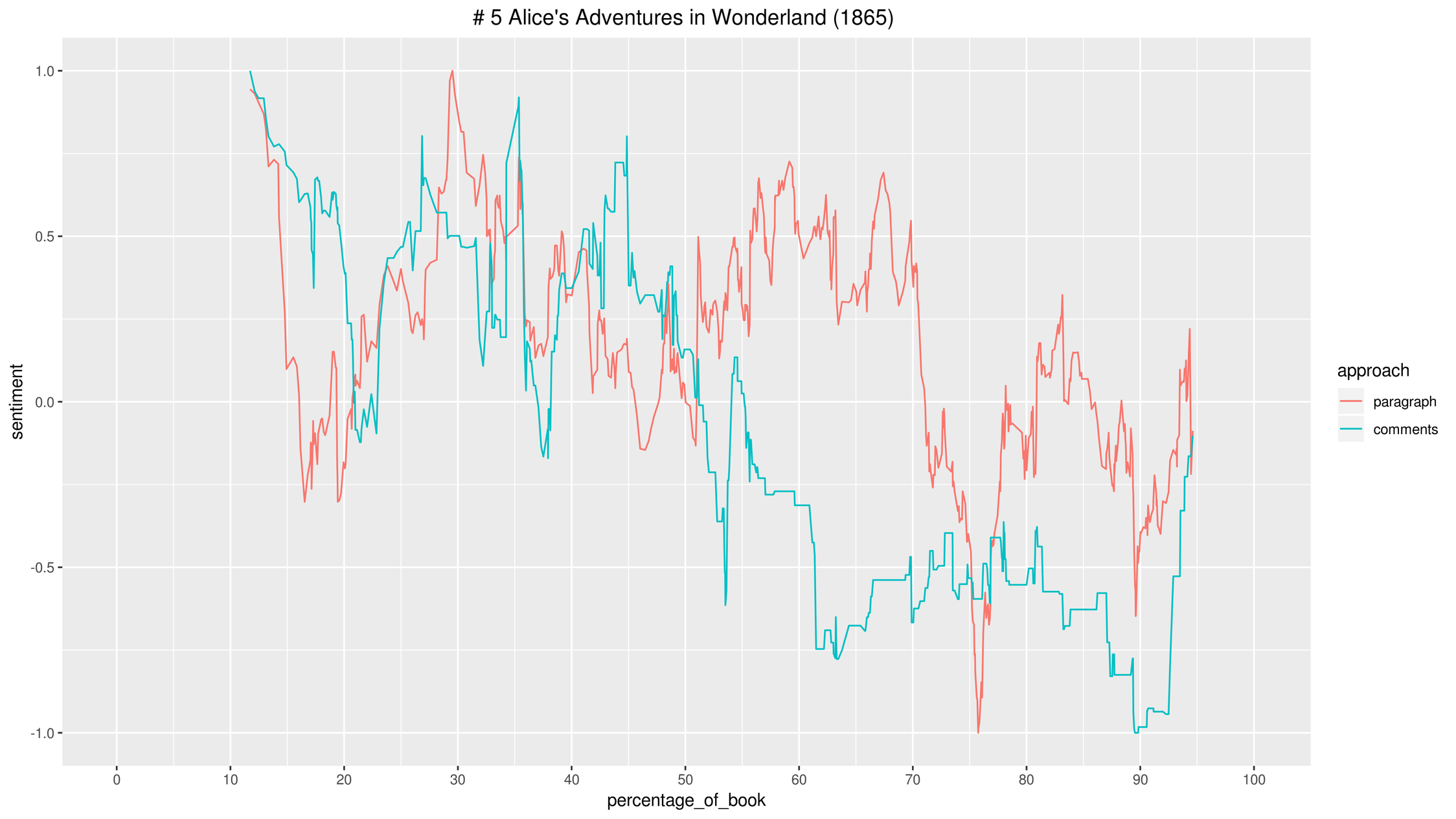

Supplement: S18 Fig — (TIF) [file pone.0226708.s023.tif]

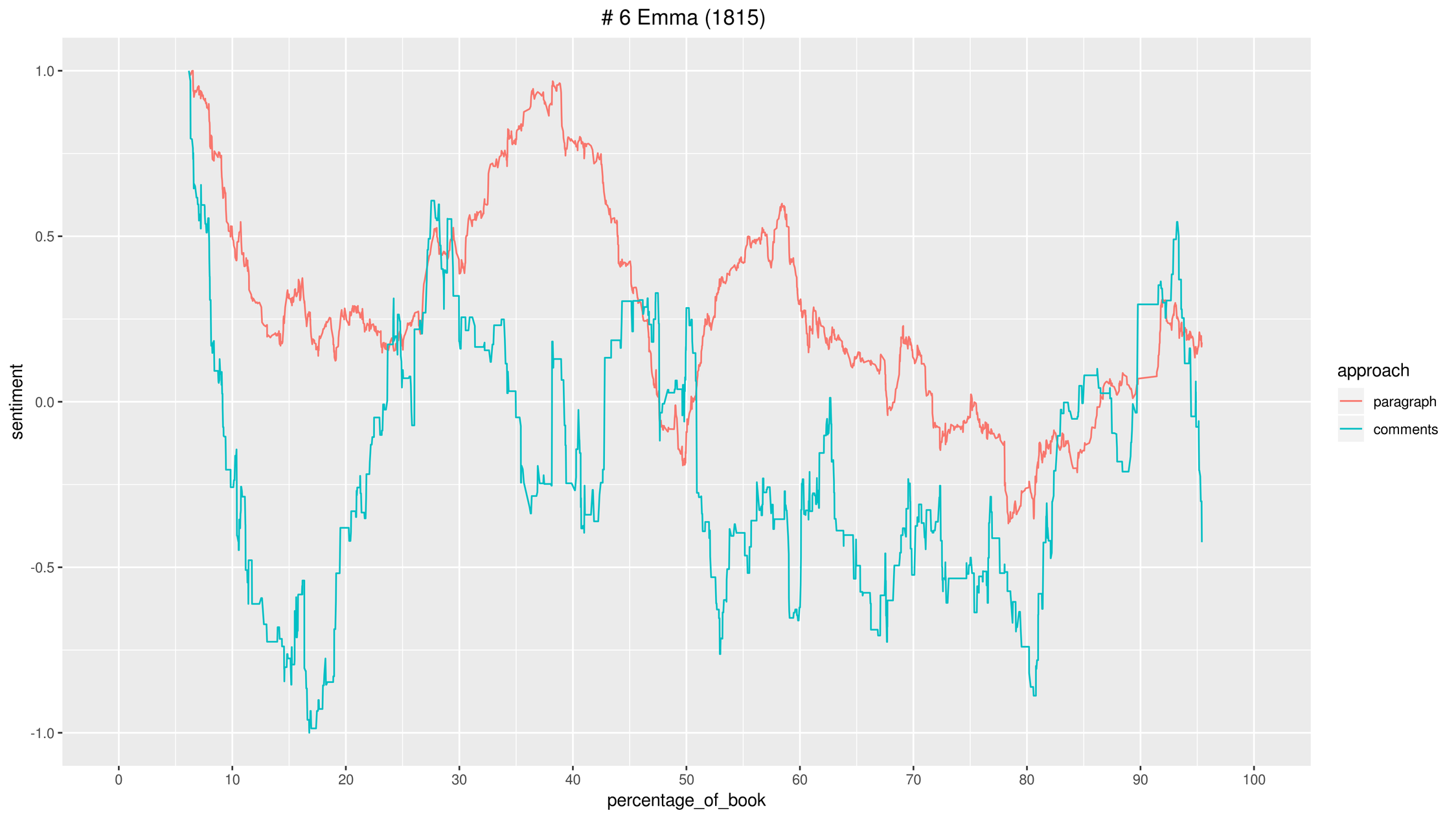

Supplement: S19 Fig — (TIF) [file pone.0226708.s024.tif]
