# Supplementary material for: Wattpad as a resource for literary studies. Quantitative and qualitative examples of the importance of digital social reading and readers’ comments in the margins
Source: PLoS One. 2020 Jan 15;15(1):e0226708. doi: 10.1371/journal.pone.0226708 (PMC6961871; doi:10.1371/journal.pone.0226708)
Supplement: Attachment — Submitted filename: Response_to_reviewers.docx [file pone.0226708.s025.docx]

**Response to the reviewers.**

1. *Please ensure that your manuscript meets PLOS ONE's style requirements, including those for file naming.*

a. We corrected the title format, a couple of missing indentations, and the position of figures’ legends.

2. C*ould you please in the revision: a) state in your methods section that you did indeed obtain permission from Wattpad to scrape the data; b) in your ethics statement, explain who you consulted, how you protected users' privacy and what other measures you took to comply with ethical standards.*

a. Done

b. Done

3. *In order to meet the requirements for the Science of Stories collection, the Guest Editors ask that you please make the code to reproduce your analysis available in a stable, public repository (for example, Zenodo, or GitHub) or a suitable cloud computing service (such as Code Ocean) when submitting your revised manuscript. The code should include a license file and detailed readme so that someone with access to the dataset is able to reproduce your analysis using the code. We ask that you include the DOI for the repository holding your code in an updated Data Availability statement with your revised manuscript.*

a. We added the R markdown DOI as S2 file in the Supplementary Information, and also added it to the Data Availability statement.

4. *We require you to either (1) present written permission from the copyright holder to publish these figures specifically under the CC BY 4.0 license, or (2) remove the figures from your submission.*

a. Copyright obtained from Flourish, Inc. for the figures.

5. Review Comments to the Author.

**Reviewer #1.**

a. *The demographic data presents the greatest challenge. The underlying point of demonstrating international appeal is clear, but how can you ensure the data is reliable. For example, I note 2 users in North Korea, which is as likely to be soft trolling as genuine users.*

This is certainly possible. In section 2.3 we already mentioned that this information is “optional and fully unstructured”, therefore acknowledging the limitation of the data. We now included a more explicit warning.

b. *My only major concern with the manuscript comes from the ethics of data mining reader data at scale. To my mind, the challenges of researching social media at scale should be addressed in the methodology or in an ethics statement (which would presumably fall under 'human subject research'). While individuals are offered pseudonyms, this may not be sufficient protection, particularly since the authors acknowledge that "data of the comments corpus cannot be shared publicly because this will expose underage users to possible identification." These are some of the trickiest issues around studying reading in 'live' online environments, and at a bare minimum it would be useful to know that this has been reviewed by the authors' institutional ethics board, but preferably this would also include further discussion in the manuscript itself.*

We added a discussion of these ethical issues in the Methods section (2.1).

c. *p. 14 - the claim that 'thanks' should be treated as a negative in the corpus would benefit from further evidence.*

Although elaborating on how to improve Syuzhet's performance in general, and on Wattpad in particular, is of importance, here we just point out some of the aspects that requires further attention. We added a better explanation of why the word “thanks” needs careful consideration when occurring in the comments.

d. *p. 41-2 the focus of the manuscript is on comments, but do likes/views across chapters confirm that most readers abandon the texts? These would be more useful metrics as they require a lower threshold for engagement*.

This is a good point. We now included views (reads) across chapters in the graphs and in the discussion.

**Reviewer #2**

a. *The chief limitation of the study is the way its near-exclusive focus on texts and reader comments brackets off considerations of production, and even of the wider import of the issues raised. In the submission’s late stages many avenues for future research are flagged but these involve a near-exclusive focus on analysing the existing or additional datasets, rather than broader issues of theoretical or commercial import. In short, the submission needs to look up from the text and data occasionally.*

Although this is a very interesting line of inquiry that will require further attention, this is a study focused on showing how Wattpad can be used as a source for studying books’ reception and readers’ response. We made this point now explicit in the introduction. We take production and distribution of texts into account inasmuch as it is related to reception, e.g. when we talk about digital-born stories and serialisation. We additionally point to a broader picture of social media writing. However, research on this issue is still scarce, thus limiting further considerations of “theoretical or commercial import” based on the data that we analysed.

*b.* *The submission lacks contextualisation of its research in reader response theory since the 1970s (classically Iser, but also landmark 1980s works such as Fish and Radway). N.B. especially recent work emerging from book history and publishing studies on readers’ uses of digital platforms (DeNel Rehberg Sedo and Danielle Fuller, Ann Steiner, Anouk Lang’s edited collection). The first of these explicitly discusses reading groups’ cross-cultural interpretations of Pride and Prejudice and hence seems highly relevant here. Lisa Nakamura’s piece for PMLA on Goodreads is also worth mentioning, especially in light of the below methodological concerns). Overall, the theoretical coverage feels thin compared to the methods section.*

We included a contextualisation in reader response theory in both the introduction and discussion sections.

Rehberg Sedo and Fuller’s books and articles offered us excellent parallels to other shared reading practices. We added references to them in the discussion section.

Lang’s edited collection is a very precious reading, we included references to it throughout the article.

Steiner’s article is about the publishing industry and not particularly relevant for the present work.

Nakamura’s article is a general invitation (much needed at the time: 2013) to study readers on Goodreads. We mentioned it in the introduction.

*c.* *Some of the material in section 3.4 seems repetitious, adumbrating minor variations in datasets or visualisations. It created a strong impatience in this humanities-trained reader to get to the actual analysis (largely delayed until p. 38’s ‘Discussion’ section). Even then, the paper suffers from a lopsidedness, with its vast methodological apparatus generating insufficient analytical pay-off or theoretical takeaways. One effect of the social-sciences focus on quantification and method is that methodologies that cast their net wider than the text itself are overlooked. e.g. the political economy of Wattpad and its commercial attempts to distil some archetypal story structure from its vast archive to drive print publication and screen adaptation deals (see https://www.theglobeandmail.com/business/article-what-clicks-with-fiction-readers-wattpad-helps-authors-find-out/ ). If such approaches are not going to be engaged with, they should at a minimum be registered and reasons for not exploring them provided.*

We here stick closely to the specificity of this PlosONE special issue “The Science of Stories”, for which this article was submitted.

As noted by Reviewer 1, we used different “data analysis techniques to triangulate the paper's main hypotheses”.

As already mentioned in point *a* above, the scope of the present article is on reading practices, not on production and publishing. A whole new article will be needed to properly cover those aspects.

*d.* *The first Simon Peter Rowberry reference [29] is incomplete. His article about Kindle Highlights would seem highly relevant but is not mentioned until p.45. See also Bronwen Thomas’ articles on fan fiction and textual interactivity, as well as her forthcoming monograph on social media and literature.*

Reference 29 has been corrected. Given the article structure required by PlosONE, we think there is no need to mention Rowberry’s article before the discussion section.

We contacted Bronwen Thomas: her “forthcoming” book is not yet drafted. Her articles about fanfiction have been useful to compare the style of comments posted on Wattpad and fanfiction sites.

*e.* *The relative paucity of readers responding to other readers (less than a third of all comments) creates the suspicion that Wattpad is a potentially interactive reading space, but that much of the actual reader commenting serves a more performative function (cf. lines1057-58). Lines 737-38 appear to reinforce this suspicion, though it is not examined in any detail. Implications of this should be teased out and tested against social reading theory relating to, for example, Goodreads.*

This is a very interesting point. We added a reflection about the performative function that is fostered by the social media context.

*f.* *There is no escaping the reality that most of the reader comments cited are notable mostly for their banality (e.g. character judgements about Pride and Prejudice). A later comment that a certain passage in a teen fiction ‘sparks a great discussion’ bases this estimation on the number of high-sentiment words it involves, not on the interpretive rigour or ingenuity of such reader comments. This raises the question of whether amateur readers like Wattpad’s users are more worth studying than expert readers such as literary critics, whose professional skills elucidate ‘hidden’ or underexamined innovative readings of existing texts. What exactly do the researchers aim to elucidate by examining amateur readers’ responses at scale if it is not the content of their comments? Granted, high-school English teachers might take heart that digital platforms create reader engagement with classic texts, even in antiquated forms of English, but would any teacher award high marks for the levels of readerly comprehension and analysis quoted in the submission?*

We strongly reject the claims that it is worthier to study literary critics, that the only goal of research on literature is to “elucidate ‘hidden’ or underexamined innovative readings”, and that teaching literature is an activity oriented to grading students’ comprehension and analysis. First, we believe that tools like Wattpad can help to develop a passion for reading, which is a very valuable quality. Second, studying reading practices doesn’t have to serve the purpose of better explaining literary texts. Third, all readers deserve respect for their reading skills, and understanding the way they read could actually highlight why literature is meaningful to them, not what they can do for literature. It seems to us that this reviewer’s comments actually reiterate the moralising dismissal of women reading groups already exposed by Anna Kiernan (“The Growth of Reading Groups as a Feminine Leisure Pursuit: Cultural Democracy or Dumbing Down?” In *Reading Communities from Salons to Cyberspace*, edited by DeNel Rehberg Sedo, 123–39. Palgrave Macmillan, 2011).

More specifically, “*by examining amateur readers’ responses at scale*” we can set the ground for statistically valid claims about how people read. Namely, we can understand what kind of emotions they feel towards the characters and how they react to specific narrative strategies like suspense. Some of the comments we presented in this article show that readers’ responses are often contradictory and by no means they can be exemplified by the literary critics’ reception of the text. Ultimately, this kind of data is also valuable to better understand the role that factors like “experiential background” (Caracciolo, Marco. *The Experientiality of Narrative: An Enactivist Approach*. Berlin: de Gruyter, 2014) and “personal relevance” (Kuzmičová, Anežka, and Katalin Bálint. ‘Personal Relevance in Story Reading : A Research Review’. *Poetics Today* 40, no. 3 (2018): 429–51) play in reader response.

*g.* *The authors’ point about the ‘feuilleton-like’ serial publishing format driving commenting frequency for teen fiction is legitimate, but overlooks the fact that many 19thC classic Anglo-American works were published serially, if not the Bronte and Austen works most commented upon in Wattpad (cf. Dickens, Thackeray).*

In this article we are only talking about Classics’ reception by 21st century readers and reading practices on Wattpad, where Classics are uploaded all-at-once. However, the parallel is interesting, so we included a reference to this historical precedent.

*h.* *In summary, the submission would benefit from lifting its gaze from the specifics of the study methodology and the Wattpad texts themselves to analyse more of the production and consumption dimensions of Wattpad, specifically the firm’s monetisation of readerly affect, and the theoretical implications (and limitations) of analysing amateur readers’ digital interactions.*

This reviewer has given us some excellent reading suggestions to improve our article. However, we think their feedback is biased by assumptions that threaten the plurality of approaches and methodologies of research on literature and reading. We have the impression that this reviewer is exclusively interested in issues related to production, especially in relation to the commercial aspects of publishing and the commodification of reading practices. We acknowledge that these are interesting issues to be examined, but we deliberately set them aside to the extent that they were not directly relevant for our research questions.

**Reviewer #3**

*a.* *Although I acknowledge that there is a significant body of literature referred to here, it seems to me not always the right or relevant literature. For example, there is a whole field of study around manuscript annotations that should be referred to beyond Kerby-Fulton et al (2012). Heather Jackson's Marginalia, for example. Bonnie Mak's work could be useful here too.*

Jackson’s books have been a precious suggestion, we referred to them in several points.

*b.* *Similarly, reader response theory is a broad field that requires more than a single reference to Bortolussi and Dixon (2003) - an empirical study - to avoid misrepresenting the field.*

We now better contextualised our study within the reader response tradition.

*c.* *I suggest Katherine Bode's work in “Reading by Numbers” and “A World of Fiction”. And, hesitatingly, I also point out my own article "Social Reading: The Kindle’s Social Highlighting Function and Emerging Reading Practices" just for interest in particular because it discusses readers commenting on Pride and Prejudice, though within the Kindle platform rather than the Wattpad platform.*

We added references to Bode’s work, where relevant, and discussed the reviewer’s article with respect to our findings.

*d.* *Social reading isn't limited to online spaces (though it is facilitated by them) and the two shouldn't be conflated. The authors use 'literary stories' but I think they mean fiction (rather than getting into the debate about what is and isn't literary)*

We made the distinction between “social reading” and “digital social reading” explicit. We corrected “literary stories” with “fiction”.

*e.* *I don't know of the use of stop words as a verbal phrase as it is used here (line 219). As far as I am aware, it is a noun phrase that would need another verb to make the sentence grammatically correct.*

We corrected it.
