# Supplementary material for: Wattpad as a resource for literary studies. Quantitative and qualitative examples of the importance of digital social reading and readers’ comments in the margins
Source: PLoS One. 2020 Jan 15;15(1):e0226708. doi: 10.1371/journal.pone.0226708 (PMC6961871; doi:10.1371/journal.pone.0226708)
Supplement: Attachment — Submitted filename: Response_to_reviewers.docx [file pone.0226708.s026.docx]

**Response to the reviewers.**

*Reviewer #3: I have two further questions the authors might like to consider:*

1. *Where is the evidence that wattpad stories are *written* on mobile phones?*

We added a reference to the Wattpad blog post in which this is stated by the company.

2. *What role might platform recommendations have played (ie if you read this you might like to read this book) in associations between texts?*

It is possible that there are different dynamics involved in increasing the popularity of a book, including platform recommendations. However, since the algorithms regulating the promotions of stories are unknown, it is difficult to assess how relevant automated recommendations are in comparison to books selected and promoted by Wattpad editors. Moreover, in the comments readers often refer to other stories they have read. In order to reliably evaluate how the associations between texts are emerging, a study focusing on network analysis will be required. For instance, using data on titles and characters extracted from the comments through name entity recognition techniques; or asking it directly to readers through questionnaires.

3. *In addition, the manuscript will require some more editing for English expression. For example, the less/fewer error, reader response vs readers' response (sometimes one or the other is required)*

We copyedited the article again and corrected the errors.
